# Immunogenicity of Potential CD4^+^ and CD8^+^ T Cell Epitopes Derived From the Proteome of *Leishmania braziliensis*

**DOI:** 10.3389/fimmu.2019.03145

**Published:** 2020-02-14

**Authors:** Rafael de Freitas e Silva, Beatriz Coutinho de Oliveira, Ailton Alvaro da Silva, Maria Carolina Accioly Brelaz de Castro, Luiz Felipe Gomes Rebello Ferreira, Marcelo Zaldini Hernandes, Maria Edileuza Felinto de Brito, Osvaldo Pompílio de-Melo-Neto, Antônio Mauro Rezende, Valéria Rêgo Alves Pereira

**Affiliations:** ^1^Department of Natural Sciences, Universidade de Pernambuco, Garanhuns, Brazil; ^2^Department of Immunology, Fundação Oswaldo Cruz, Recife, Brazil; ^3^Parasitology Laboratory, Universidade Federal de Pernambuco, Vitória de Santo Antão, Brazil; ^4^Department of Pharmaceutical Sciences, Universidade Federal de Pernambuco, Recife, Brazil; ^5^Department of Microbiology, Fundação Oswaldo Cruz, Recife, Brazil

**Keywords:** neglected diseases, cutaneous leishmaniasis, *Leishmania (Viannia) braziliensis*, immunogenicity, CD4^+^ CD8^+^ T cell epitopes

## Abstract

**Background:** A safe and effective vaccine against human leishmaniasis still requires the identification of better antigens for immunization and adequate models to evaluate the immune response. To support vaccine development, this work tested the immunogenicity of 10 different peptides derived from the proteome of *Leishmania braziliensis*, which were selected by their *in silico* affinity to MHC complexes.

**Research design and Methods:** Comparative cell proliferation assays were performed by culturing, in the presence of each peptide, PBMC cells from subclinical subjects (SC), cutaneous leishmaniasis patients with active disease (AD), post-treatment (PT) individuals, and healthy controls. Culture supernatants were then used for Th1, Th2, and Th17 cytokine measurements. Cells from selected PT samples were also used to assess the expression, by T cells, of the T-bet Th1 transcription factor.

**Results:** A robust cell proliferation was observed for the SC group, for all the tested peptides. The levels of Th1 cytokines were peptide-dependent and had substantial variations between groups, where, for instance, IFN-γ and TNF levels were some of the highest, particularly on PT cultures, when compared to IL-2. On the other hand, Th2 cytokines displayed much less variation. IL-6 was the most abundant among all the evaluated cytokines while IL-4 and IL-10 could be found at much lower concentrations. IL-17 was also detected with variations in SC and AD groups. T-bet was up-regulated in CD4^+^ and CD8^+^ T cells from the PT group after stimulation with all peptides.

**Conclusions:** The peptide epitopes can differentially stimulate cells from SC, AD, and PT individuals, leading to distinct immune responses.

## Introduction

The leishmaniasis are an important group of neglected tropical diseases (NTDs) (1, 2) caused by protozoans of the genus *Leishmania*. These diseases are endemic in 98 countries and territories, with an annual incidence of 1.5–2 million cases and with at least 350 million people living in areas with a higher transmission risk. Clinical manifestations may occur under different forms, and they can be broadly classified as visceral (VL) and cutaneous (CL) leishmaniasis (3). In humans, VL is more limited in number of cases, although it is associated with a higher lethality. In contrast, CL is more widespread and responsible for afflicting a more substantial number of individuals. Although this disease is usually relatively benign, it may cause disfigurement and loss of productivity (4). VL is mainly caused by *L. donovani* and *L. infantum*, while many different *Leishmania* species are involved in the development of CL, such as *L. braziliensis* (in the New World) and *L. major* (Old World). Currently, alternatives to reduce these diseases rely solely on vector control and by using old and toxic antimonial drugs, for which there is an increasing number of resistant parasites (5). Thus, there is an urgent need for a safe and effective vaccine against leishmaniasis to be applied in humans.

The evidence that a vaccine against CL can be achievable is based on the fact that the recovery from the infection with *L. major*, and to some extent with *L. braziliensis*, may induce protection against reinfection (6, 7), suggesting that natural infection is capable of inducing a protective immunity (8). So far, however, there are no licensed vaccines for human CL and those that advanced to clinical trials include only two killed *L. amazonensis* vaccines (9–11). This may be due to the fact that there are missing points in the knowledge regarding immunity against *Leishmania* and which might be misleading vaccine development. Contributing to that, most of the acquired data come from murine models using *L. major*, which does not necessarily correlate with *L. braziliensis*. We also do not fully understand the immune response against *Leishmania* in humans, or how to generate memory against reinfection and what are the best correlates of protection to look for (8, 12). Moreover, one of the main challenges facing the development of a vaccine is the identification of the best type or set of antigens to correctly induce immunity against *Leishmania*, since traditional whole parasite vaccines have major safety concerns (13, 14).

Different cell subsets and molecules mediate the immune response against *Leishmania* infection. The cellular immune response starts with the priming of naïve T lymphocytes by dendritic cells (DCs), capable of presenting *Leishmania* peptides bound to major histocompatibility complex molecules (MHCs)/human leukocyte antigens (HLA). These cells, generally dispersed throughout the body, recirculate in the blood and lymph and can co-stimulate, produce, and secrete cytokines to mount a T cell response (15). Immunity against *Leishmania* is achieved through the development of T CD4^+^ and T CD8^+^ lymphocytes producing key cytokines (16). For murine models, the T CD4^+^ Th1 profile is implicated with parasite clearance and disease control while a Th2 profile is associated with a high parasite burden and disease progression (17). However, for humans, the response is more complex and contributions from different profiles may be required to achieve a specific response. For example, the interleukin IL-17 (Th17) has been implicated with a massive influx of inflammatory cells and disease exacerbation (18) and IL-10 (Th2) has been implicated with strong immunosuppression and exacerbated pathology (19). Therefore, it is possible that distinct cytokines from different sources can also act together to induce a protective immune response (20, 21).

The activation of CD4^+^ T cells is a result of the specific engagement of 15-mer peptides bound to MHC II, while CD8^+^ T cells are activated by 9-mer peptides to MHC I molecules. It is estimated that each DC can express 10^6^-10^7^ MHC Class II (MHC II) and 10^5^ MHC Class I (MHC I) proteins (22). Considering the potential of *L. braziliensis* antigens and the lack of successful vaccines and approaches for their development, we have previously described the use of a combination of *in silico* methods in the search for potentially robust CD4^+^ and CD8^+^ T cell epitopes in the proteome of *L. braziliensis* (23). We have described a set of 15-mer peptides with an intrinsic potential to bind MHC complexes from major alleles present in human populations. The top 10 ranked peptides were capable of significantly stimulating the proliferation of immune cells from post-treatment CL patients. In this context, the aim of this work was to analyze the *in vitro* immunogenicity induced by these peptides on cells from leishmaniasis patients, grouped according to the clinical stage of the disease: subclinical (SC), active disease (AD), and post-treatment (PT). All peptides were capable of stimulating cell proliferation in PBMCs from the selected samples and induced the expression of most of the quantified cytokines, with variations, however, that depended on the groups from which these cells were isolated and on the peptides. Relevant results were identified based on these differences.

## Patients and Methods

### Study Design and Ethics Statement

Patients and individuals were selected from both genders with a median age of 33 (ranging from 18 to 60) who came from the municipality of Moreno, state of Pernambuco, Brazil. Samples were clustered into four different groups: subclinical (SC, *n* = 10), corresponding to those who lived in endemic regions, positive for the Leishman Skin Test (LST), and who had no history of clinical disease; active disease (AD, *n* = 8), who were diagnosed patients with CL; post-treatment (PT, *n* = 13), patients treated for up to 1 year and who were considered clinically cured; and one group of healthy individuals living in non-endemic regions for leishmaniasis, considered as negative controls (*n* = 5). The research protocol was evaluated and approved by the Human Research Ethics Committee from IAM/FIOCRUZ (Protocol number 522.964) and all individuals have signed the “Term of free and informed consent.” Patient characteristics are related in Table 1.

**Table 1:** Patient characteristics.

**Patient**	**Sex**	**Age**	**Number of lesions**	**Clinical form**	**Evolution (months)**	**Direct search**	**PCR**	**Culture**	**Montenegro reaction**
1	M	59	1	Ulcerated	6	ND	+	ND	NR
2	M	40	1	Ulcerated	7	ND	+	ND	NR
3	M	19	2	Ulcerated	1	ND	+	ND	+
4	M	23	1	Ulcerated	1	ND	+	ND	+
5	F	21	1	Ulcerated	2	+	+	+	NR
6	M	48	1	Ulcerated	3	+	+	+	NR
7	M	24	3	Ulcerated	1	–	+	–	NR
8	F	46	1	Ulcerated	1	ND	+	+	NR
9	M	18	5	Ulcerated	3	ND	+	+	NR
10	M	53	2	Ulcerated	6	+	+	+	NR
11	M	20	1	Ulcerated	2	+	+	+	NR
12	F	27	1	Ulcerated	3	+	+	–	NR
13	F	18	1	Vegetative	3	–	–	+	NR
14	F	34	1	Vegetative	3	+	+	+	NR
15	F	20	1	Vegetative	2	+	+	+	NR
16	M	19	5	Ulcerated	2	–	+	+	NR
17	F	60	1	Ulcerated	3	–	+	+	NR
18	M	45	2	Ulcerated	2	+	+	-	NR

*ND, non-determined; NR, non-reagent*.

### Synthetic Peptides and Storage

Molecules corresponding to the previously described top 10 15-mer peptides (23) were commercially synthesized (Genome Biotechnology, Brazil). Linear peptides were purified through high-performance liquid chromatography (HPLC), with a final purity >95%. All the synthetic peptides were individually resuspended in dimethyl sulfoxide (DMSO) and stored at −80°C until further use.

### Isolation and Culturing of Human PBMCs

From each individual enrolled in this research, 20 ml of blood was aseptically collected in heparin tubes, diluted 1:1 (v/v) in phosphate buffered saline (PBS, pH 7.2), and deposited into a Ficoll-paque PLUS (GE Healthcare, USA) gradient solution, followed by centrifugation at 400 × g for 35 min. The peripheral blood mononuclear cell (PBMC) layer was then removed and washed twice with PBS. The cells were then resuspended in RPMI 1640 medium containing 2 mM L-glutamine, 50 mg/L gentamicin sulfate, and supplemented with 10% fetal bovine serum (from Cultilab reagents, Brazil). The resuspended cells were counted, and their concentration was adjusted to 10^6^ cells/ml, with 2 × 10^5^ cells then deposited per well in 96-well U-bottom plates (BD Falcon, USA). Each well was individually stimulated with 20 μg/ml of peptide and then the plates were incubated at 37°C in 5% CO_2_ for 4 days. The plates were subsequently centrifuged for 10 min at 400 × g and the supernatant was collected and stored at −80°C for cytokine measurement by flow cytometry. The assays were performed in triplicate and each peptide was evaluated separately.

### CFDA-SE Labeling and Cell Culture

Cells from AD, PT [data derived from E Silva et al. (23)], SC, and the control groups were labeled with CFDA-SE (carboxyfluorescein diacetate succinimidyl ester, Invitrogen, USA) in order to assess the level of cell proliferation induced by the peptides. The PBMCs (4 × 10^6^) were resuspended in 1 ml of PBS supplemented with 2 μM of CFDA-SE and incubated at 37°C for 10 min (the CFDA-SE concentration was previously titrated in order to prevent inhibition of cell proliferation or cell death). Following incubation, the labeling was quenched with 1 ml of ice-cold (4°C) RPMI 1640 supplemented with 2 mM of L-glutamine, 50 mg/L of gentamicin sulfate and 10% fetal bovine serum. The cells were pelleted and washed with PBS followed by resuspension in 1 ml of RPMI 1640 supplemented to a density of 2 × 10^6^ cells/ml. The PBMCs were plated in 96-well U bottom plates in triplicate at a density of 2 × 10^5^ cells/well, with 20 μg/ml of each peptide, followed by incubation at 37°C with 5% CO_2_ for 96 h. The cells were then removed from the plates, deposited in polystyrene tubes, washed with PBS, and analyzed by flow cytometry. For each patient or control, non-stimulated and 20 μg/ml phytohemagglutinin (PHA)-stimulated labeled cells were evaluated as intra-experimental controls to set the level of cell proliferation during the flow cytometry data analysis.

### Th1, Th2, and Th17 Cytokine Measurements

Cell supernatants from SC, AD, PT, and control groups were thawed and the measurement of the IFN-γ, IL-2, IL-10, IL-4, TNF, IL-6, and IL-17a cytokines was performed using kits for human Th1, Th2, and Th17 cytometric bead arrays (CBA) (Cytometric Bead Array kit, BD Biosciences). All methods were carried out according to the manufacturer's recommendation. The samples were analyzed by flow cytometry on a FACSCalibur flow cytometer (Becton Dickinson Company, San Jose, USA). For each sample, 2100 bead events were acquired as recommended by the manufacturer. The standard curve for the CBA assay was determined using nine dilutions, and the data were analyzed with the FCAP Array software, provided by BD.

### Anti-T-Bet Cell Labeling

Cells from four PT individuals and four controls were used to evaluate the levels of T-bet expression. After peptide stimulation and subsequent washing with PBS-W (PBS plus 0.5% bovine serum albumin and 0.1% sodium azide—both from Sigma, St. Louis, MO), 6 × 10^5^ cells were transferred to polystyrene tubes and incubated for 30 min at room temperature with monoclonal antibodies anti-CD4 and anti-CD8, conjugated with allophycocyanin (APC) and fluorescein isothiocyanate (FITC), respectively (both from BD Biosciences, San Jose, CA). The cells were then fixed with 1% paraformaldehyde (PFA) in PBS, washed with PBS-W, and permeabilized with PBS plus 0.5% saponin. Following new washes with PBS-W, they were subsequently incubated with phycoerythrin (PE)-conjugated anti-T-bet (BD Biosciences, San Jose, CA) for 30 min, at 25°C. They were washed again with PBS-W and resuspended in PBS with 1% PFA. Leishmania total lysate antigen was used in most of the experiments (data not shown) and induces as stronger cytokine and proliferative response compared to short peptide-epitope sequences tested in this work.

### Flow Cytometry Analysis of CFDA-SE and T-Bet-Labeled Cells

All flow cytometry analyses were performed on a FACSCalibur flow cytometer (Becton Dickinson Company, San Jose, USA) equipped with an argon laser (wavelength 488 nm) and using the CELLQuestPro™ software (BD Biosciences, San Jose, CA) for cell signal acquisition. Fluorescence of 20,000 lymphocyte gated events, based on scatter parameters of size and granularity, was acquired. The data were analyzed and treated with FlowJo v10.1 (Tree Star Inc., USA). For the CFDA-SE assay, non-stimulated cells were used during the analysis to set quadrant parameters and the basal level of lymphocyte proliferation. For the T-bet assay, labeled cells from control individuals were used to set the quadrant standards.

### Heat Map, Data Clustering, and Statistical Analysis

The data for the median levels of IFN-γ, TNF, IL-2, IL-6, and IL-17 expression were used to create a heat map and clustered using an algorithm with package in R. Hierarchical dendrograms represent the degree of relatedness among the data.

The statistical analysis was performed using the GraphPad Prism Software v.7 and the data were analyzed with the non-parametric Mann–Whitney *U*-test. The differences were considered statistically significant when *p* ≤ 0.05.

## Results

### Description of the Peptides Selected for This Study

The 10 peptides investigated here were restricted to human MHC supertypes and were mapped to four different proteins from *L. braziliensis*, which are conserved among different *Leishmania* species. Eight peptides are derived from three hypothetical proteins: four from LbrM.34.3630 (peptides 1, 4, 5, and 8), a polypeptide found to include a zinc finger and whose *T. brucei* ortholog localizes to the cytoplasm; three from LbrM.01.0110 (peptides 6, 7, and 10), found in both *Trypanosoma* and *Leishmania* species but with yet undefined properties or other features; and one from LbrM.06.0820 (peptide 3), a hypothetical protein found only in *Leishmania* and closely related groups but so far absent from *Trypanosoma* species. The last two peptides (2 and 9) were derived from LbrM.14.1680, a protein annotated as a putative Synaptojanin, an inositol/phosphatidylinositol phosphatase involved with vesicle-mediated transport. Peptides derived from the same protein share substantial overlaps in sequence differing by one or two amino acid residues (peptides 1 and 4, for example, have 14 residues in common, as do peptides 4 and 5, all from LbrM.34.3630). Peptides from different proteins share little similarity in sequence, although all are enriched in aromatic residues. Table 2 displays all MHC/HLA alleles, which were used by the *in silico* methods to predict the peptide epitopes from the present work, and Table 3 lists all peptides evaluated here as well as the proteins from which they are derived (20).

**Table 2:** MHC/HLA allele coverage applied by the *in silico* epitope prediction methods.

**Type of prediction method**			
**Linear**		**Structural**	
**MHC I supertype**	**MHC II supertype**	**MHC I**	**MHC II**
HLA-A1	HLA-DPA	HLA-B5703	HLA-DR3
HLA-A2	HLA-DPB	HLA-B*1501	HLA-DR2 (DRA*0101,DRB1*1501)
HLA-A3	HLA-DQA	HLA-B*2709	HLA-DRA1*0101/DRB5*0101
HLA-A24	HLA-DRB	HLA B*4405	HLA-DQ2
HLA-A26		HLA-B*4402	HLA-DQ0602
HLA-B7		HLA-A*0301	HLA-DR2b
HLA-B27		HLA-B7	HLA-DQ8
HLA-B44		HLA B*3508	HLA-DRA, DRB3*0101 (DR52a)
HLA-B58		HLA-B*57	HLA DR52c
		HLA-A*2402	HLA-DP2
		HA B2705	HLA-DQ1
		HLA-A68	HLA-DP5
		HLA-B*44:03	
		HLA-A*1101	
		HLA-B*51:01	
		HLA A*0101	
		HLA-B*3901	
		HLA B*3501	
		HLA B*0801	
		HLA-A24	
		HLA-B*1801	

**Table 3:** List of 15-mer peptides evaluated in this study.

**Peptide**	**Peptide sequence**	**Protein ID**	**Characteristics**
P1	FLYYYILCYARDFGS	LbrM.34.3630	Hypothetical protein, conserved
P2	GNDHYYEYILWKYHG	LbrM.14.1680	Synaptojanin (N-terminal domain), putative
P3	ISFEIYPAHLFYSLI	LbrM.06.0820	Hypothetical protein, conserved
P4	PFLYYYILCYARDFG	LbrM.34.3630	Hypothetical protein, conserved
P5	APFLYYYILCYARDF	LbrM.34.3630	Hypothetical protein, conserved
P6	VFYTISFDQMERYLA	LbrM.01.0110	Hypothetical protein, conserved
P7	YTISFDQMERYLAAI	LbrM.01.0110	Hypothetical protein, conserved
P8	HNAPFLYYYILCYAR	LbrM.34.3630	Hypothetical protein, conserved
P9	NDHYYEYILWKYHGA	LbrM.14.1680	Synaptojanin (N-terminal domain), putative
P10	TVFYTISFDQMERYL	LbrM.01.0110	Hypothetical protein, conserved

### CFDA-SE Cell Proliferation Assay

To evaluate whether the peptide epitopes would stimulate PBMCs from active disease (AD) and subclinical (SC) individuals, we performed the CFDA-SE proliferation assay comparing those groups with healthy controls. Both sets of AD and SC PBMC samples were first found to be capable of proliferating when stimulated with total antigen from *L. (V.) braziliensis*, as previously described by our group for post-treatment CL (PT) patients (21), with non-stimulated cells having minimal levels of proliferation (data not shown). For the analyses with the different peptides, representative histograms for the flow cytometry analysis of one SC individual are shown in Figure S1. For these histograms, the internal labeled control and non-stimulated samples were used to set the gates that were applied for all the other samples tested with the peptides. This step was performed for each sample. Generally, the PBMCs from all individuals in the different groups were tested with all 10 peptides, but for the SC group, the analysis with peptide 5 was only possible with two individuals.

**Figure 1:**
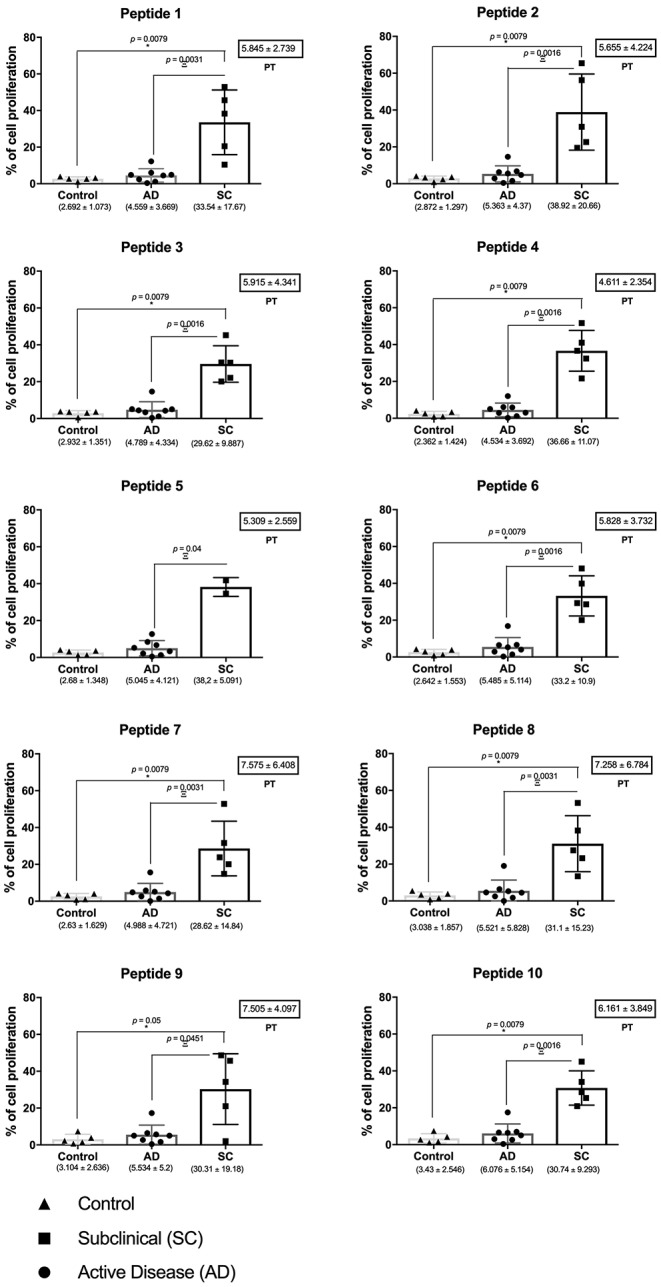
Peptide epitopes tested induce high levels of proliferation by cells from subclinical individuals for cutaneous leishmaniasis. Evaluation of the CFDA-SE proliferation assay for cells from healthy controls as well as individuals from the active disease (AD) and subclinical (SC) groups. Horizontal bars represent the medium values, and their standard deviations, calculated from the evaluation of eight AD, five SC, and five control individuals. The values are presented bellow each graph, with the black dots representing individual values. For comparison, the mean proliferation values and standard deviations for the post-treatment (PT) group [derived from reference (23)] are shown in the small boxes on the left. The asterisk represents the significant differences between SC and control group, and the Greek letter Ξ (ksi) shows the significances between SC and AD groups. Significant differences were considered when *p* ≤ 0.05.

The results from the assays carried out with the PBMCs from the whole set of AD, SC, and control individuals are shown in Figure 1. The data from the synthetic peptides are presented and the mean percentage of proliferation, with its standard deviation, was calculated with the results from the proliferation of PBMCs derived from eight AD and five SC individuals, when exposed to the individual peptides. For the AD samples, the mean AD proliferation values ranged from 4.5 to 6%, and none of the peptides were capable of significantly stimulating cell proliferation. Indeed, the overall proliferation profile is similar to that seen for the healthy control samples, although the mean proliferation values are slightly higher than those from the control group (2.4–3.4%). In contrast, for the SC group, all peptides could significantly stimulate PBMC proliferation with mean values ranging from 28.6 to 38.9%. These values are not only substantially increased when compared with the AD individuals in this study, but are also considerably higher than the values reported for the PT group, performed in our previous work (23). Indeed, it is possible to observe that PBMCs from all five SC individuals showed high levels of proliferation for almost all the tested peptides. PHA-stimulated and non-stimulated cells were used as positive and negative control, respectively, to determine the gates, and it is not displayed in the graphs.

### Th1 Cytokine Measurements

**Figure 2:**
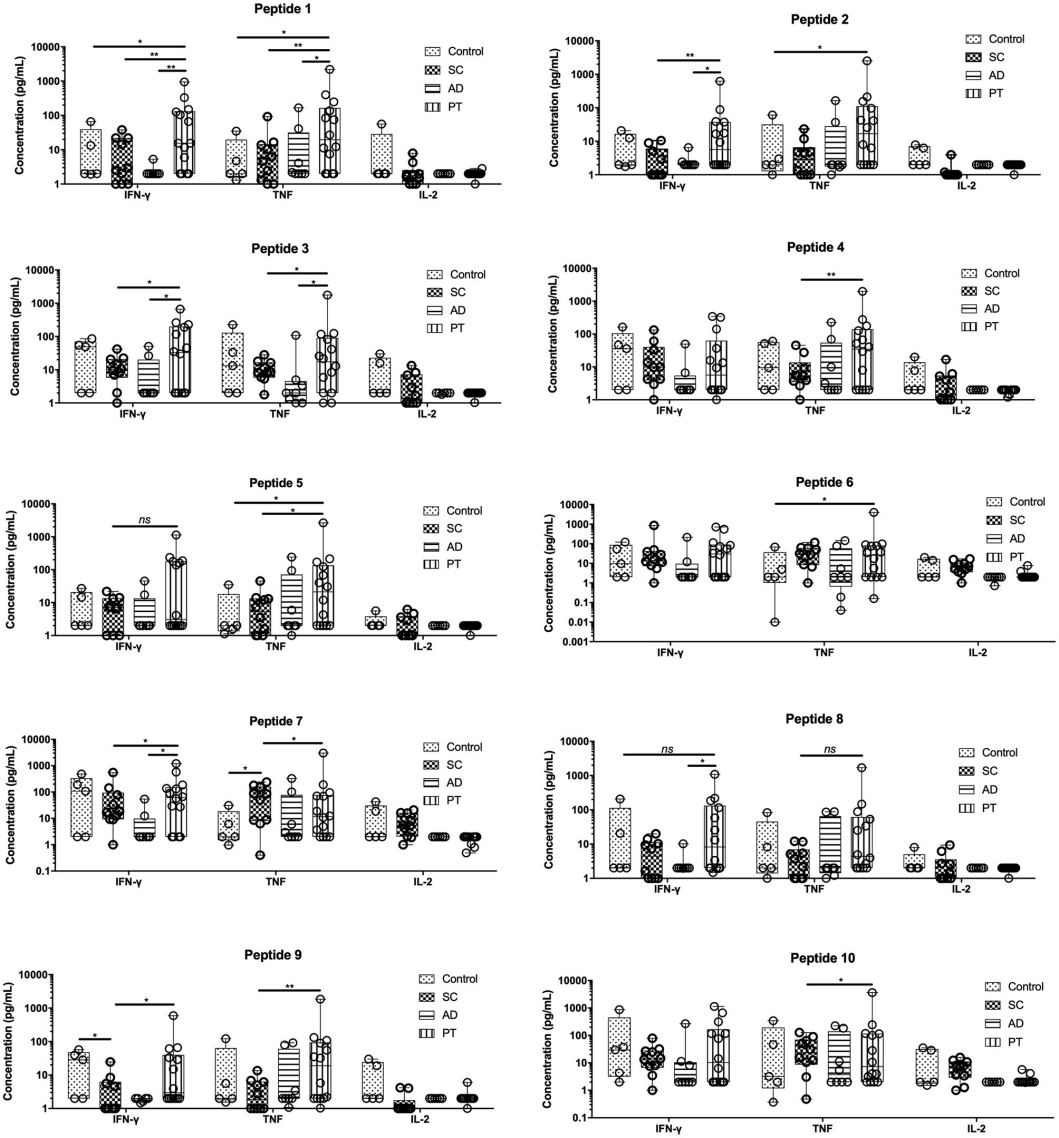
PBMCs from post-treated individuals for cutaneous leishmaniasis when stimulated with peptides produce significant levels of IFN-γ and TNF. Measurement of IFN-γ, TNF, and IL-2, in the supernatant of cells from control individuals as well as sub-clinical (SC), active leishmaniasis (AD), and post-treatment (PT) patients. The bars represent the medium values and their standard deviations, calculated based on the data from the cell supernatants from 14 PT, 8 AD, and 5 healthy control individuals. Significant differences were considered when *p* ≤ 0.05, *p ≤ 0.05, **p ≤ 0.005; *ns*, non-significant.

The levels of three cytokines associated with the Th1 profiles, IFN-γ, TNF, and IL-2, were first measured with the goal of evaluating the cytokine response secreted in the cell culture supernatant after PBMC stimulation (Figure 2). The results are represented based on the mean cytokine concentration values, in pg/ml, plus their standard deviations after peptide stimulation. These were calculated based on the data from the cytokine measurement within the culture supernatants from 5 controls, 10 SC, 8 AD, and 13 PT individuals.

The levels of IFN-γ and TNF were generally the highest in the PT group when compared to the other groups for most of the peptides tested, with mostly statistically significant differences. For IFN-γ, exceptions were seen for peptides 4, 6, 7, and 10 in which the levels of these cytokines were approximately the same between PT and control groups (peptides 4, 7, and 10) or between the control and SC groups (peptide 6). Low levels of IFN-γ were generally found for the AD group, for many of the tested peptides, but these were particularly low for peptides 1, 2, 8, and 9 (<3 pg/ml). Regarding TNF levels, the major differences in its production was found for the PT individuals when compared to the AD and SC group. Differently from the IFN-γ in which the AD group generally secreted low levels of this cytokine, the AD group secreted higher levels of TNF, for example, when compared with the control individuals. For the third cytokine investigated, IL-2, low levels were generally verified in the supernatants tested. Its expression profile, however, was somewhat reversed when compared with the two other Th1 cytokines, with higher levels observed in control individuals and on occasions for the SC group as well. Indeed, the highest levels of this cytokine for the SC group were seen with peptide 6, 7, and 10, all derived from the same hypothetical protein (LbrM.01.0110). In terms of relative percentage contribution of each Th1 cytokine produced (Figure S2), there is an important production of TNF by cells from AD and PT individuals, after the stimulation with these peptides and this contribution is even more pronounced for individual PT cells. Moreover, comparatively among the non-control groups, there is an important contribution of IL-2 by SC cells. Paradoxically, however, the cells from the SC group responded well proliferating, but failed to significantly induce the production of Th1 and, for that, low levels were detected in this group compared to PT cells. The overall data are consistent with distinct profiles induced by these last three peptides when compared with the remaining peptides derived from the three other proteins cited in this study.

### Th2 Cytokine Measurements

**Figure 3:**
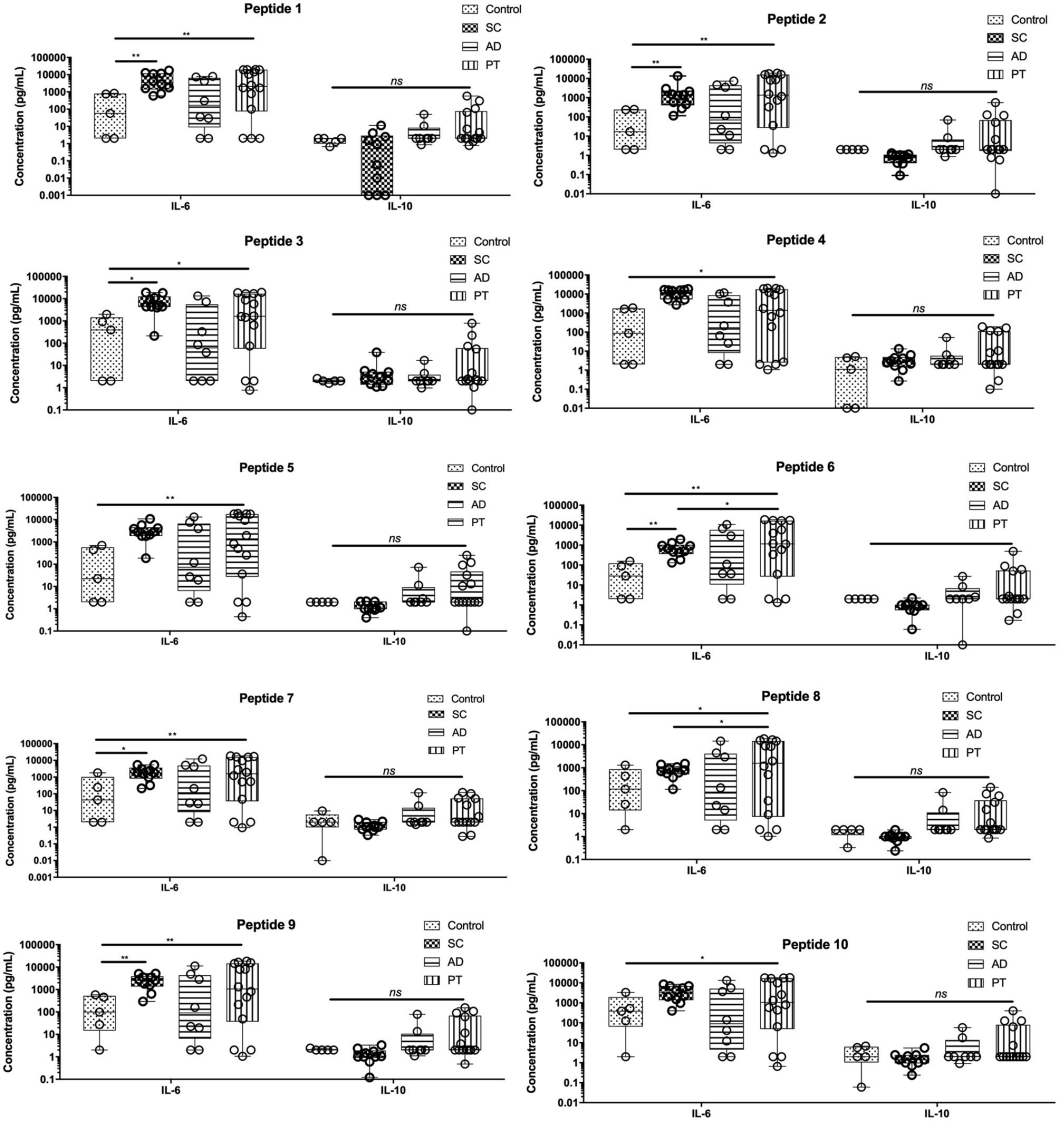
Peptide epitopes induce the secretion of IL-6 by PBMCs of subclinical and active disease and of IL-6 and IL-10 by cells from post-treated leishmaniasis individuals. Measurement of IL-10 and IL-6 levels in the supernatant of cells from control individuals as well as sub-clinical (SC), active leishmaniasis (AD), and post-treatment (PT) patients. The bars represent the medium values and their standard deviations, calculated based on the data from the cell supernatants from 14 PT, 8 AD, and 5 healthy control individuals. Significant differences were considered when *p* ≤ 0.05, *p ≤ 0.05, **p ≤ 0.005; *ns*, non-significant.

Three cytokines that are mainly associated with the Th2 profile (IL-4, IL-6, and IL-10) were also evaluated in cell culture supernatants of PBMCs from the same individuals of all the tested groups (Figure 3). Low levels of IL-4 were detected for all samples when compared to the other cytokines and no major differences in its production were observed between groups or peptides (all cytokine data are related in Table S1). In contrast, IL-6 was the only cytokine that had its production and secretion significantly stimulated by all the peptides investigated in this work. Lower levels of IL-6 were found on the control group for all the tested peptides, but were generally higher in supernatants from cells derived from the PT group. Overall, the levels of IL-6 were very similar for the groups SC, AD, and PT, with little or no differences observed between the various peptides. Regarding IL-10, for all peptides, its levels were the highest among PT and AD groups, having lower levels in control samples and, in most cases, the same was observed for the SC group. In general, for the Th2 cytokines, no relevant association between peptides or their original proteins could be related to their production profile.

### Th17 Cytokine Measurements

**Figure 4:**
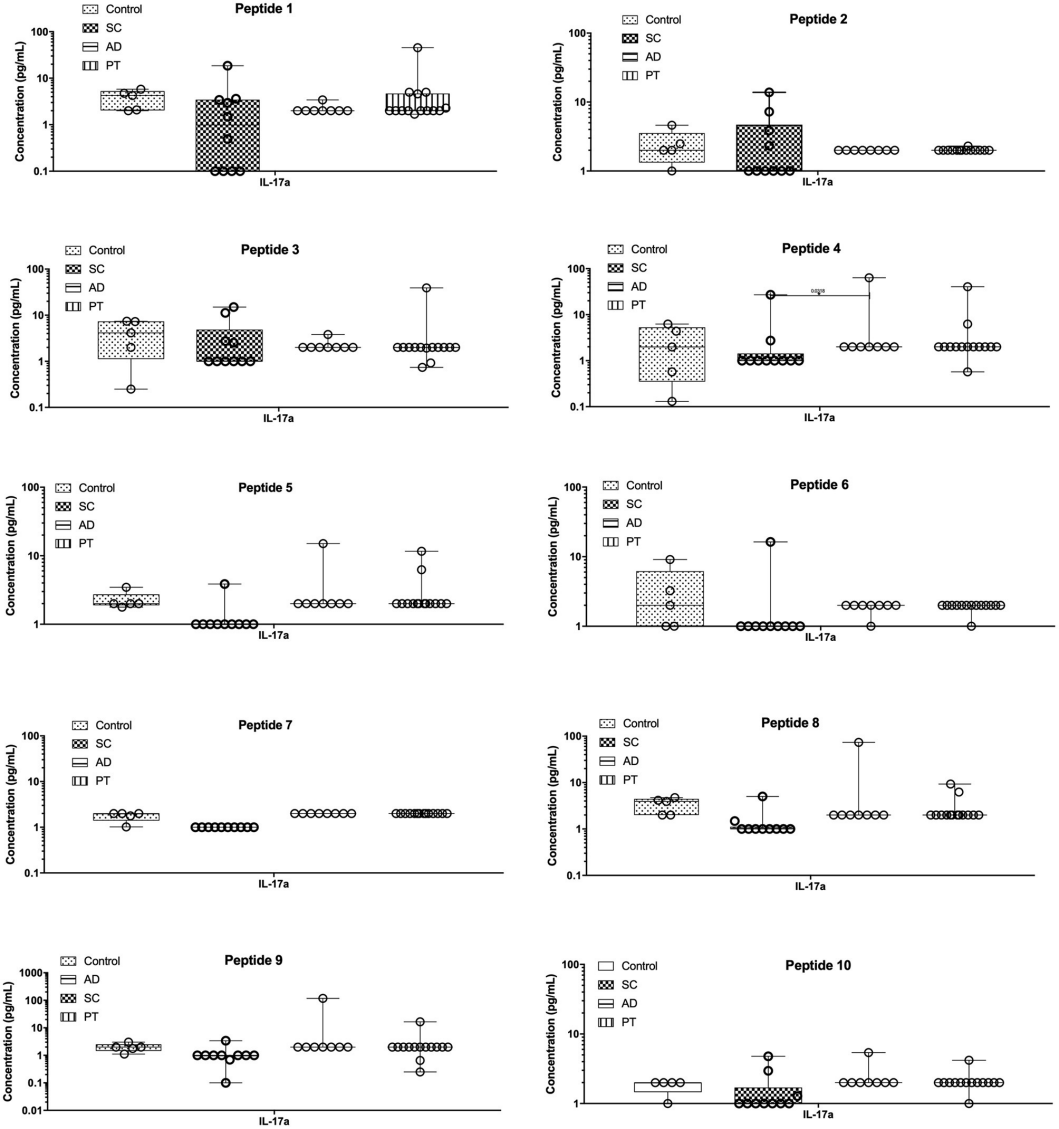
The epitopes tested induced very low levels of pro-inflammatory cytokine IL-17. Measurement of IL-17a levels in the supernatant of cells from control individuals as well as sub-clinical (SC), active leishmaniasis (AD), and post-treatment (PT) patients. The bars represent the medium values and their standard deviations, calculated based on the data from the cell supernatants from 14 PT, 8 AD, and 5 healthy control individuals.

The Th17 (IL-17a) profile was measured as described (Figure 4). After stimulating PBMCs with the different peptides, low levels of IL-17a were detected for all groups, with minor differences observed in control individuals or those from the PT group. Regarding the AD group, however, two distinct profiles were generated by peptides 4, 8, and 9, as they were capable of inducing substantially higher concentrations of IL-17a when compared to the remaining peptides and also higher than those from the other groups that were exposed to the same peptides. In contrast, for the SC group, peptides 5, 8, and 9 produced lower levels of IL-17a when compared with the other groups, resulting in more than 10-fold differences for peptides 8 and 9 between AD and SC groups. It is noteworthy that there are related effects induced by peptides 4, 5, and 8, all derived from the same *L. braziliensis* hypothetical protein (LbrM.34.3630).

### Cytokine Heat Map and Data Clustering

**Figure 5:**
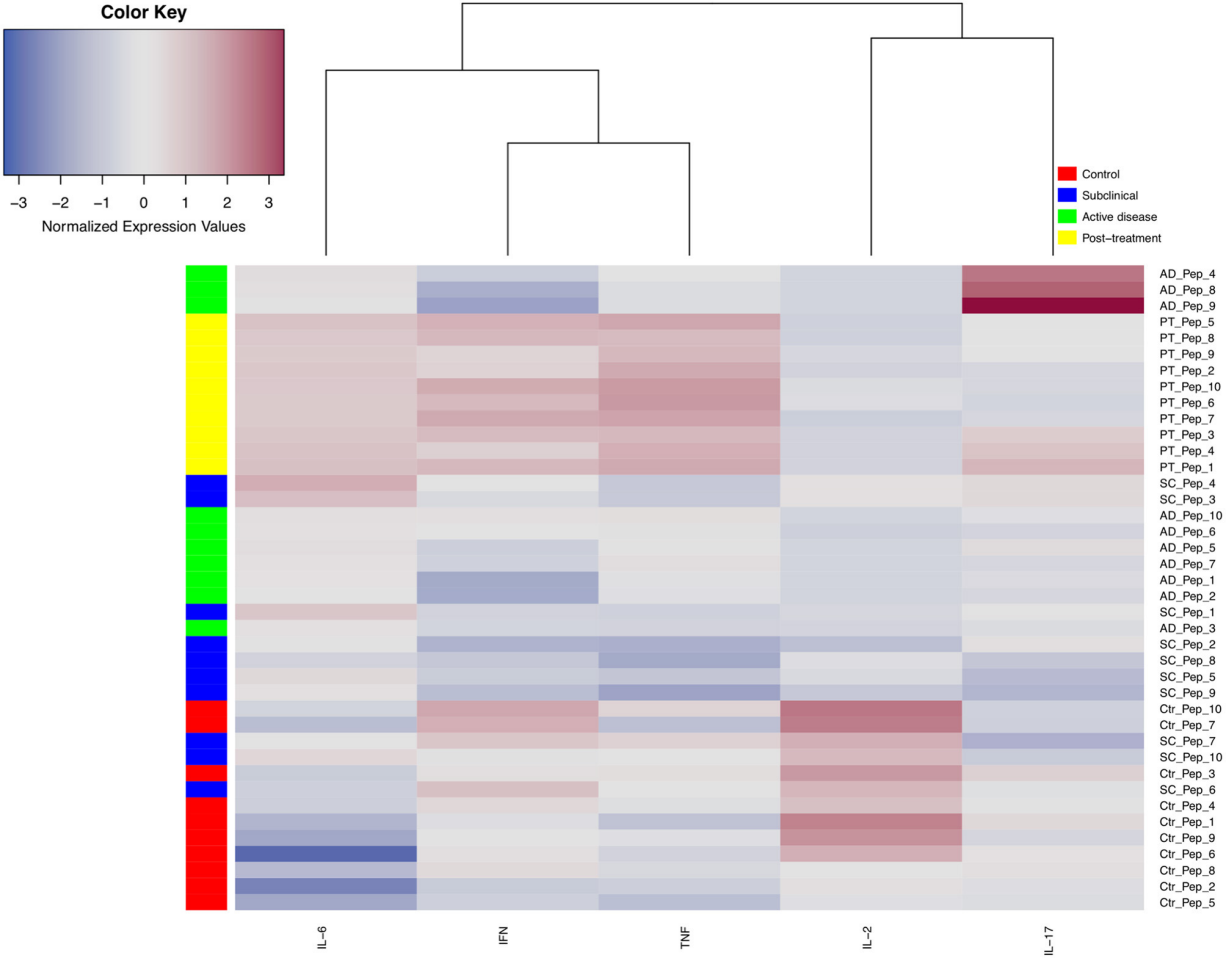
Heat map and cluster analysis of cytokine profiles secreted by PBMCs from different groups of leishmaniasis individuals when individually stimulated with 10 different peptide epitopes. The vertical cluster represents the grouping of individuals considering 10 peptides (Pep), from Pep_1 to Pep_10. The horizontal cluster displays the grouping of five cytokines, IL-6, IFN-γ, TNF, IL-2, and IL-17a. Normalized cytokine expression values are indicated using a color scale, ranging from blue (low) through gray to red (high). Different colors also represent the groups assessed (control = red, subclinical = blue, green = active disease, yellow = post-treatment). The horizontal dendrogram above the heat map displays degrees of relatedness between the cytokine profiles investigated.

To produce an overview of the cytokine response for all peptides from the different groups, a cytokine heat map was created consolidating the data based on the median expression levels from the five cytokines that gave more significant results, IFN-γ, TNF, IL-2, IL-6, and IL-17 (Figure 5). By applying this analysis, it is possible to observe that the main cytokines stimulated by the peptides (red color) were IFN-γ, TNF, and IL-6, produced mainly by PT cells. Indeed, the horizontal dendrogram on the top of the heat map confirms that those three cytokines belong to a cluster due to the similar responses observed. In contrast, as represented in the heat map, IL-2 is mainly produced by the control and AD samples, while IL-17 is generally poorly induced although higher levels were detected after stimulation of AD and/or PT cells with peptides 1, 3, 4, 8, and 9.

### T-Bet^+^ Cell Expression

**Figure 6:**
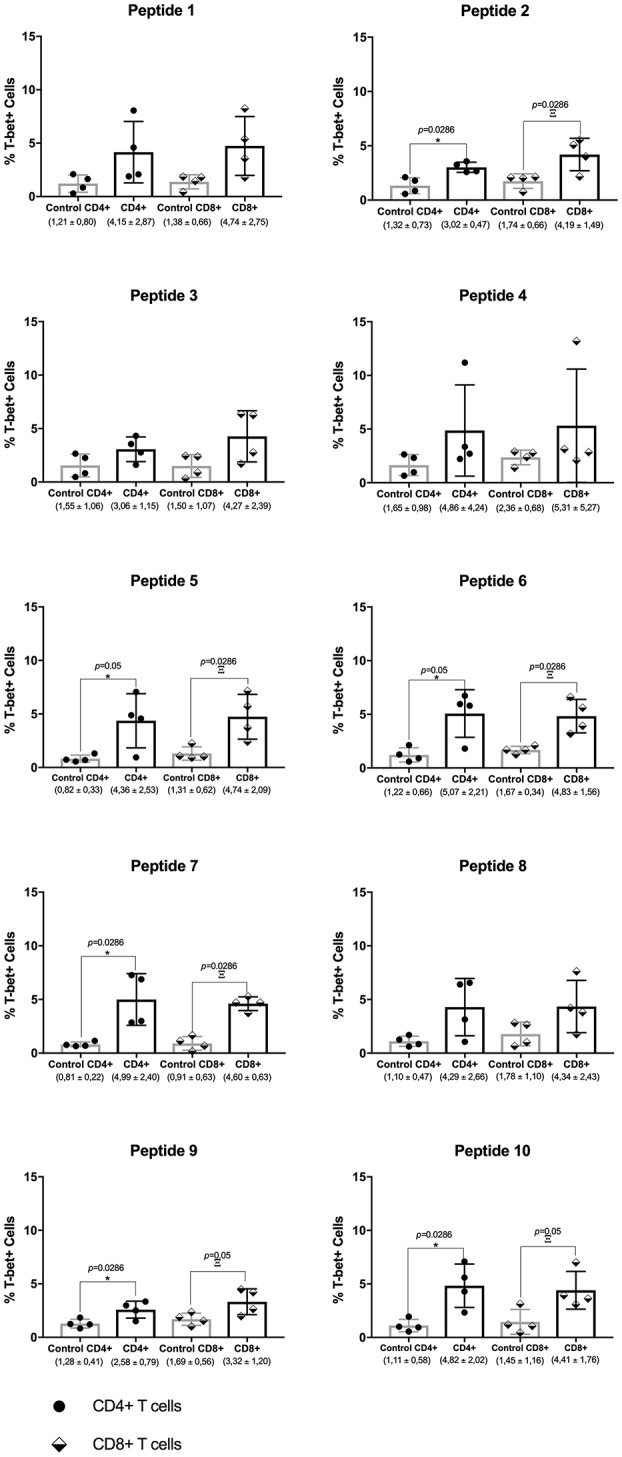
Th1 transcription factor master regulator T-bet is produced by CD4^+^ and CD8^+^ T cells from post-leishmaniasis treatment individuals. Evaluation of T-bet expression in CD4^+^ and CD8^+^ T cells from post-treatment (PT) and control individuals. The horizontal bars represent the mean values calculated for each group. The black arrows indicate the values obtained with the analysis. The asterisk represents the significant differences between T CD4^+^ cells between the control individuals and those from the PT group, with the Greek letter Ξ (ksi) showing the significances between T CD8^+^ cells from the same groups. The median percentage levels and the corresponding standard deviation for each group tested are described below. Significant differences were considered when *p* ≤ 0.05.

For the PT group, a general trend from the cytokine data is the increased levels of various cytokines after peptide stimulation. This was seen for most peptides, after comparing the data with cells from the other groups, and it was mainly seen for the most abundant cytokines assayed: IFN-γ, TNF, IL-6, and IL-10. This result contrasts with the cell proliferation analysis where the SC group showed much higher levels of proliferation when compared with the PT individuals and other groups. In this sense, focusing on a more detailed analysis of the effect of the chosen peptides on selected samples, T-bet expression was analyzed in CD4^+^ and CD8^+^ T lymphocytes by flow cytometry after peptide stimulation in PBMCs. T-bet (encoded by *Tbx21*) is a transcription factor that acts as the master regulator of Th1 profile commitment (21). A representative assay with dot plots for T-bet expression analysis with cells from one PT individual is shown in Figure S3. For each individual, above the main population of cells, gates were settled with labeled controls. Four PT individuals were compared with four healthy controls, and the data for each peptide are summarized in Figure 6. An increased level of T-bet expression in both T CD4^+^- and T CD8^+^-stimulated cells was observed for all peptides, with most of these being statistically significant. In contrast, minimal levels were achieved for control counterparts. For the PT group, the mean levels of T-bet ranged from 2.58 to 5.07% for T CD4^+^ and from 3.32 to 5.31% for T CD8^+^ cells. For the control group, the medium levels of T-bet ranged from 0.8 to 1.65% for T CD4^+^, and from 0.9 to 2.36% for T CD8^+^ cells. These results demonstrate that T-bet was up-regulated for both types of lymphocytes in the PT group.

## Discussion

In this study, we have demonstrated that the 10 peptides retrieved from *L. braziliensis* proteome by *in silico* methods can specifically stimulate cell proliferation, cytokine production, and secretion, as well as the activation of the Th1 transcription factor T-bet. Differences were observed in the profiles induced by the different peptides, which also varied according to the groups evaluated here. It is noteworthy that there were both different cell proliferation responses and contrasting patterns of cytokine expression induced by the groups AD, SC, and PT. It is also relevant that the distinct expression pattern of the Th1 cytokines in response to the peptides can be correlated with the protein from which they were derived. These results highlight aspects of the immune response against *L. braziliensis* infection that can be further investigated using specific peptides that are able to generate different cytokine expression patterns.

During the cell proliferation analysis, it was possible to observe that the SC group was the most responsive to the peptides, suggesting that these individuals have memory T cells that are capable of recognizing the epitopes. This observation agrees with previous results (23) obtained with PBMCs from PT patients, which showed greater levels of proliferation when compared to the control. The degree of cell proliferation observed here might be a direct consequence of the number of T cell clones present in the secondary lymph organs. We suggest that this proliferation response observed for the SC group is a consequence of the number of mature T cells circulating in these individuals, since, in theory, they are boosted by the persistence of parasites in lymphoid organs and re-infections (22). Moreover, the expansion of T cells is a consequence of how these antigens are presented and how the cells are activated (23). The development of memory T cells is one of the most important aspects that are desired for a vaccine (24), and previous works have suggested that the induction of memory T cells is a critical step for the development of an anti-*Leishmania* vaccine (25, 26). The proliferative response observed in the present work might indicate the recall of these cells by the presence of the peptides. The lack of response by cells from control individuals or the AD group suggests the absence of memory T cells that could recognize any of the epitopes tested. One of the main roles of memory T cells during reinfection is to produce key cytokines that act against the pathogen (27). The differences observed in responses stimulated by the peptides might be related to their intrinsic capacity to bind the MHC molecules expressed on the surface of APCs or even their susceptibility to APC processing and presentation to T cells (28, 29).

Regarding the cytokine analyses, the most revealing results pointed out here were from the Th1 cytokines, IFN-γ, TNF, and IL-2, although in many cases a substantial response was also observed for the control cells. Indeed, most of the tested peptides were capable of inducing the expression and secretion of IFN-γ and TNF, and it has been known for a long time that IFN-γ and TNF may act together in order to promote a Th1 profile that induces macrophage activation with nitric oxide (NO) production, culminating with *Leishmania* killing (30, 31). Although the Th1 immune profile has an important influence on the control of *Leishmania* infection, it has also been implicated in its pathogenesis. It has been described in murine models and also in humans, that high levels of TNF are associated with more severe forms of CL and chronicity, and the use of TNF inhibitors along with antiparasitic drugs have shown more promising disease outcomes (32–35). Contradictory to what was expected, cells from SC subjects failed to mount a Th1 immune response upon peptide stimulation despite their higher proliferative capacity. Those findings are very interesting and suggest that a complex network of cells and cytokines may play an important role on this. In an endemic population for *L. braziliensis* in Bahia (Brazil), cells from SC individuals were found to produce low levels of TNF and IFN-γ compared to those with CL (36). Interestingly, it was reported that regulatory T cells (Tregs) from SC patients had an improved capacity to suppress IFN-γ production than Tregs from CL patients (37). Furthermore, hypo/non-responsiveness to primary vaccines has been also correlated with higher percentage of Tregs and B regulatory cells (38–40). The evidence from this study suggests that these SC subjects might be constantly exposed to *Leishmania* antigens and developed a delay type response characterized by lower levels of IFN-γ and higher levels of IL-6 and IL-10.

As for the Th2 cytokines, both IL-6 and IL-10 were produced after stimulating cells with the peptides in different groups, and substantially increased levels were observed in the three groups evaluated when compared to the controls. IL-6 has been involved with the induction of different T cell profiles, such as Th1, Th2, Th17, and Tregs (41). The levels of IL-6 induced by the peptides may have a dual role as pro-inflammatory cytokine and also preventing regulatory T cell profiles, which might be associated with disease exacerbation (42, 43). For an ideal vaccine against CL, it is expected that long-lasting memory T cells are induced, with an anti-*Leishmania* response mediated by the Th1/Th2 immunobalance (36, 37). The presence of IL-10 is important since it has been shown in humans that it is one of the main cytokines that have been associated with immunosuppression and pathology in CL (38, 39). Thus, the induction of IL-10 altogether with cytokines such as IFN-γ and TNF may counterbalance the immune response.

T-bet is the master regulator of Th1 commitment, and its role in resistance against some intracellular pathogens was already confirmed (40). Nevertheless, the role of T-bet in *Leishmania* infection is not yet clear, but the results from the present study suggest that the peptide epitopes can induce its expression. In addition, since the peptides used were chosen based on their ability to stimulate both CD4^+^ and CD8^+^ T cells, this result confirms that both subsets of T cells have been shifted toward a Th1 profile. T-bet is only expressed in the absence of negative stimulation such as that provided by TGF-β (44). This result thus corroborates the possibility that some of the peptide epitopes may have a potential to be recognized and induce a Th1 response against *Leishmania* infection.

The results presented here overcome important limitations during vaccine research. One of these is the validation process that is performed with murine models, and this may not reflect what happens with human cells (45–47). Animal models of leishmaniasis are limited since the immune response they develop cannot be translated to humans. These limitations reinforce the importance of performing validation approaches using human samples, which may translate with more confidence what would be expected in the human body. In addition, previous research show results from peptides that are derived from known proteins or which were previously evaluated as a whole antigen, thus narrowing the possibilities to find new potential antigens (48, 49). *In silico* tools can be important to speed up the search for new potential antigens that could trigger the immune response against pathogens (50). The cell proliferation values observed in our study for the SC group strongly suggest that there is a potent T cell presentation and recognition with the samples from these individuals. Furthermore, the recognition of those epitopes might be correlated with the development of T cells that are capable of recognizing and reacting against those peptides from *L. (V.) braziliensis* (51). Our results then validate the use of the *in silico* tools for the identification of novel antigens and peptides that can potentially be used as part of a candidate vaccine against human cutaneous leishmaniasis.

## Conclusions

All peptides tested here have the ability to significantly induce the proliferation of cells from subclinical individuals, but not from those with active disease or healthy controls. Also, most of the peptides were capable of stimulating the secretion of IFN-γ, TNF, and IL-6 in the supernatant of PBMC cultures. The expression of T-bet by CD4^+^ and CD8^+^ T cells was induced from all the tested groups with variations and they can be related to the different groups and peptides that presented distinct immune responses.

## Data Availability Statement

The datasets generated for this study are available on request to the corresponding author.

## Ethics Statement

This study was carried out in accordance with the recommendations of Human Research Ethics Committee from Instituto Aggeu Magalhaes/FIOCRUZ with written informed consent from all subjects. All subjects gave written informed consent in accordance with the Declaration of Helsinki. The protocol was approved by the Protocol number 522.964.

## Author's Note

Part of the results presented in this article are published in RS's thesis (Universidade Federal de Pernambuco, Brazil), which is in line with the author's university policy, and can be accessed online (52).

## Author Contributions

RS designed the idea and experiments and wrote the paper. RS, BO, and AS performed the experiments with human samples. MBre helped with cytokine analyses. LF, MH, and AR designed peptide epitope selection tools. MBri recruited patients. OM-N contributed with the idea and wrote the paper. VP contributed with reagents and data interpretation and wrote the paper.

### Conflict of Interest

The authors declare that the research was conducted in the absence of any commercial or financial relationships that could be construed as a potential conflict of interest.
