# Supplementary material for: Immunogenicity of Potential CD4+ and CD8+ T Cell Epitopes Derived From the Proteome of Leishmania braziliensis
Source: Front Immunol. 2020 Feb 14;10:3145. doi: 10.3389/fimmu.2019.03145 (PMC7033680; doi:10.3389/fimmu.2019.03145)
Supplement: Supplementary file 1 [file Data_Sheet_1.pdf]

Supplementary Material

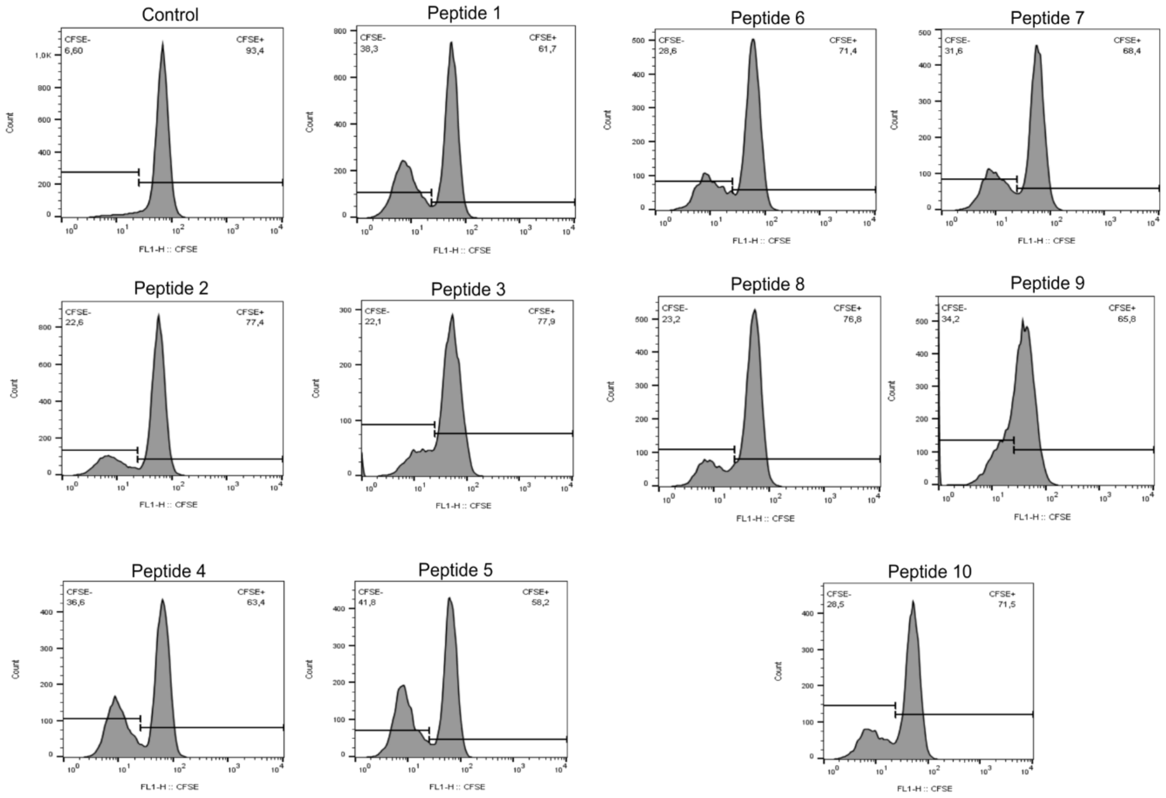

Suppl. Fig. S1: Cell proliferation by CFSE in labelled human PBMC stimulated with peptides. Histograms are representative staining for CFSE in PBMC from one SC individual.

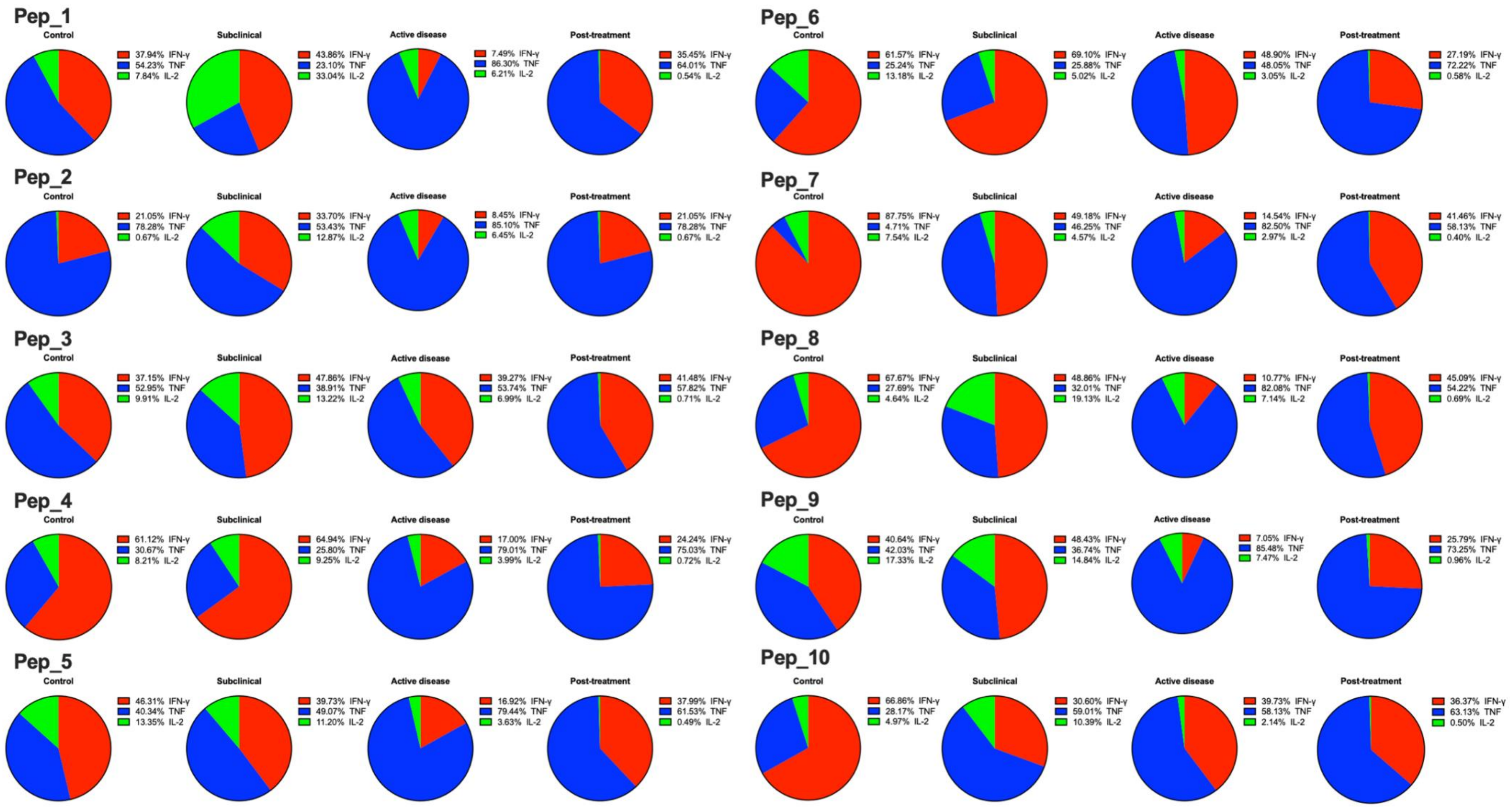

Suppl. Fig. S2: Relative contribution of Th1 cytokines produced after individually stimulation with peptides per group of individuals. The pie charts illustrate the relative percentage contribution of IFN-γ, TNF and IL-2 (Th1 cytokines) from culture supernatant of PBMC from different groups of individuals after individual stimulation with peptide epitopes.

Suppl. Tab. 1: Detailed information on individual measurements of secreted cytokines upon PBMCs stimulation with peptides.

Test Peptide 1 – Cytokines [pg/mL]

	**CT** (*n*=5)					**SC** (*n*=10)									
**IL-17a**	2,08	4,26	4,74	2	5,82	0	0	0	3,63	1,48	3,41	0,49	18,54	2,96	0
**IFN-γ**	2	2	13,32	65,8	1,93	2,5	0	14,39	2,94	37,93	21,91	2,02	0	21,91	0
**TNF**	2	2	4,71	34,79	1,29	16,52	1,31	6,21	2,45	92,03	13,87	4,67	0	10,33	0,7
**IL-10**	2	2	0,66	1,31	2	4,1	0	0	0,93	2,5	11,38	0	0,06	1,56	0,01
**IL-6**	2	2	829,64	56,62	754,03	17543,38	2827,2	1781,83	1582,02	11152,64	781,13	12823,2	596,55	11790,55	4172,19
**IL-4**	2	2	2	2	2	0	0	0	0	0	0	0	0	0	0
**IL-2**	2	2	2	56,08	2	1,66	1,5	1,9	1,99	1,9	7,93	0,23	0	4,29	0

Test Peptide 1 – Cytokines [pg/mL]

	**AD** (*n*=8)								**PT** (*n*=14)													
**IL-17a**	2	2	2	2	2	2	3,42	2	2	2	2	2	126,18	11,7	104,46	150,14	941,38	328,74	5,97	15,57	15,57	66,47
**IFN-γ**	2	2	5,3	2	2	2	2	2	2	2	2	2	7,5	137,13	84,28	27,1	2192,82	75,13	11,28	249,14	12,16	398,49
**TNF**	2	2,18	167,17	2	2	40,85	4,09	2	2	2	2	2	0,77	1,31	6,97	4,4	573,11	107,77	2	2	65,67	301,53
**IL-10**	2	2	50,03	2	2	10,44	0,86	2	98,96	2	2,05	2	786,45	1714,42	10495,14	14359,68	19332,7	19767,76	1684,99	2440,73	19332,7	19989,19
**IL-6**	29,45	300,39	4178,27	36,02	2	7317,14	7876,8	2	2	2	2	2	2	2	2	2	2	2	2	2	2	2
**IL-4**	2	2	2	2	2	2	2	2	2	2	2	2	2	2	2,85	2	2	0,22	2	2	2	2
**IL-2**	2	2	2	2	2	2	2	2	2	2	2	2	126,18	11,7	104,46	150,14	941,38	328,74	5,97	15,57	15,57	66,47

Test Peptide 2 – Cytokines [pg/mL]

	**CT** (*n*=5)					**SC** (*n*=10)									
**IL-17a**	2,49	0,65	4,62	2	2	1	1	1	2,32	1	13,85	7,22	3,86	1	1
**IFN-γ**	2	2	1,76	20,88	12,5	1	1	8,9	5,08	10,54	1	1	1	2,94	1
**TNF**	2	2	0,57	60,75	3,01	4,88	1,37	4,38	23,25	11,92	0,99	0,4	1	4,78	0,08
**IL-10**	2	2	2	2	2	1,05	1	0,37	0,74	0,4	1,3	0,09	1	1,13	1
**IL-6**	2	2	236,27	17,19	245,85	2934,18	447,36	277,15	576,94	1280,25	13376,15	1389,58	116,73	2133,96	1508,94
**IL-4**	2	2	2	2	2	1	1	1	1	1	1	1	1	1	1
**IL-2**	2	2	2	7,85	6,3	1	1	0,64	3,95	1	1	1	1	1,19	1

Test Peptide 2 – Cytokines [pg/mL]

	AD (*n*=8)								PT (*n*=14)													
**IL-17a**	2	2	2	2	2	2	2	2	2	2	2	2	2	2	2	2	2	2,3	2	2	2	2
**IFN-γ**	2	2	2,43	2	2	6,52	2	2	2	2	2	2	88,2	16,59	17,5	9,32	620,52	38,84	2	2	2	37,43
**TNF**	2	0,39	164,04	2	2	36,86	1,7	2	2	2	2	2	7,96	96,83	25,8	41,32	2536,36	42,74	2	155,04	6,35	210,7
**IL-10**	2	2	69,23	2	2	8,29	0,86	2	2	2	2	2	0,77	0,58	0	6,7	556,49	119,89	2	2	51,53	127,7
**IL-6**	11,06	116,63	3485,92	22,68	2	7240,67	4679,15	2	35,74	2	1,32	2	737,77	1161,02	8130,26	8571,96	17691,79	16198,27	333,04	1540,45	15846,23	18907,
**IL-4**	2	2	2	2	2	2	2	2	2	2	2	2	2	2	2	2	2	2	2	2	2	2
**IL-2**	2	2	2	2	2	2	2	2	2	2	2	2	2	2	2	2	2	0,95	2	2	2	2

Test Peptide 3 – Cytokines [pg/mL]

	CT (*n*=5)					SC (*n*=10)									
**IL-17a**	4,15	7,29	7,42	2	0,25	1	1	2,53	1	2,74	15,06	1	11,24	1	1
**IFN-γ**	2	2	54,56	48,99	86,25	2,02	14,25	41,7	9,65	20,61	22,29	6,97	8,17	10,93	1
**TNF**	2	2	13,2	225,83	33,2	1,78	8,17	28,21	17,98	8,61	11,37	5,3	5,98	16,29	8,17
**IL-10**	2	2	1,57	2,59	2	2,11	1,09	1,17	5,01	4,18	5,74	3,11	1,43	38,55	2,44
**IL-6**	2	2	913,91	394,98	1977,04	211,88	4335,14	4390,97	3965,14	9074,86	18862,56	10847,87	4994,2	18058,56	4390,97
**IL-4**	2	2	2	2	2	1	1	1	1	1	1	1	1	1	1
**IL-2**	2	2	2	29,95	15,73	1	2,9	8,24	6,88	1,66	1	1,12	0,84	13,37	1

Test Peptide 3 – Cytokines [pg/mL]

	AD (*n*=8)								PT (*n*=14)													
**IL-17a**	2	2	2	2	2	2	2	3,81	2	2	2	2	228,88	45,5	35,15	189,25	667,2	261,17	30	2	2	116,15
**IFN-γ**	2	26,18	2	2	2	50,64	2	2	0	0	2	2	8,35	81,75	21,65	25,8	1766,88	41,32	12,9	117,02	5,88	124,34
**TNF**	1,3	3,36	0	2	0	107,88	4,99	2	2	2	2	2	1,05	2,45	2,26	4,62	784,1	72,68	0	2	55,28	223,03
**IL-10**	2	0,97	2	2	2	16,81	4,4	2	74,14	2	0,77	2	649,8	1432,53	5814,28	8663,29	18492,88	16558,63	1571,44	1651,72	16742	19332,7
**IL-6**	38,23	328,95	2	84,89	2	13306	7240,67	2	2	2	2	2	2	2	2	2	2	2	2	2	2	2
**IL-4**	2	2	2	2	2	2	2	2	2	2	2	2	2	2	1,03	2	2	2,02	2	2	2	2
**IL-2**	2	1,81	2	2	2	2	2	2	2	2	2	2	228,88	45,5	35,15	189,25	667,2	261,17	30	2	2	116,15

Test Peptide 4 – Cytokines [pg/mL]

	**CT** (*n*=5)					**SC** (*n*=10)									
**IL-17a**	0,13	1,98	4,38	0,57	6,24	1	1	2,74	1	1	27	1	1	1	1
**IFN-γ**	2	2	163,59	46,84	36,06	9,65	33,13	61,05	10,03	13,81	132,72	4,23	4,23	2,94	1
**TNF**	2	2	51,43	60,75	9,49	9,06	5,52	27,51	5,19	2,67	45,26	3,91	0,23	5,3	3,73
**IL-10**	0	0	4,41	5,19	1,11	4,42	1,71	4,18	2,44	3,11	13,08	2,16	1	6,34	0,27
**IL-6**	2	2	1620,39	86,78	1880,6	18590,29	8831,29	16800,46	5125,29	15862,82	16560,51	14981,99	2691,04	16091,75	5328,97
**IL-4**	2	2	2	2	2	1	1	1	1	1	0,49	1	1	1	1
**IL-2**	2	2	2	19,96	7,69	1,12	1,74	16,73	5,25	1	6,17	0,91	1	3,95	1

Test Peptide 4 – Cytokines [pg/mL]

	**AD** (*n*=8)								**PT** (*n*=14)													
**IL-17a**	2	2	2	2	2	2	63,32	2	2	2	2	2	7,96	66,44	51,62	29,49	1977,85	176,43	2	126,87	40,39	274,81
**IFN-γ**	2	2	6,61	2	2	49,53	2	2	2	2	2	2	0,1	0,28	10,44	8,29	191,46	107,77	2	2	119,89	164,11
**TNF**	2	3,12	226,52	2	2	69,05	9,96	2	191,92	2	1,07	2	656,69	1040,81	12201,31	9135,58	17115,24	20213,27	2,69	1736,19	19548,94	19119,01
**IL-10**	2	2	51,99	2	2	6,43	3,68	2	2	2	2	2	2	2	2	2	2	2	2	2	2	2
**IL-6**	25,95	216,08	3746,64	64,21	2	11560,52	10272,79	2	2	2	2	2	2	2	1,2	2	2	1,46	2	2	2	2
**IL-4**	2	2	2	2	2	2	2	2	2	2	2	2	7,96	66,44	51,62	29,49	1977,85	176,43	2	126,87	40,39	274,81
**IL-2**	2	2	2	2	2	2	2	2	2	2	2	2	0,1	0,28	10,44	8,29	191,46	107,77	2	2	119,89	164,11

Test Peptide 5 – Cytokines [pg/mL]

	**CT** (*n*=5)					**SC** (*n*=10)									
**IL-17a**	1,78	2	3,47	2	2	1	1	1	1	1	3,86	1	1	0,31	1
**IFN-γ**	2	2	14,34	26,79	2	1	7,21	13,38	7,09	21,72	7,56	6,49	1	14,1	1
**TNF**	2	2	1,11	34,38	1,56	1,6	0,61	14,08	13,07	7,21	45,26	3,56	1	12,1	1
**IL-10**	2	2	2	2	2	2,11	1	1,13	2,16	0,89	1,01	0,4	1	2,11	1
**IL-6**	2	2	446,7	22,68	703,48	10847,87	1846,92	2827,2	2133,96	3915,13	2862,38	4172,19	188,38	5763,15	1869,18
**IL-4**	2	2	2	0	2	1	1	1	1	1	1	1	1	1	1
**IL-2**	2	2	2	5,58	2	1	1,05	2,9	3,62	1	4,64	0,28	1,05	6,17	1

Test Peptide 5 – Cytokines [pg/mL]

	**AD** (*n*=8)								**PT** (*n*=14)													
**IL-17a**	2	2	2	2	2	2	15,05	2	2	2	2	2	160,79	4,11	178,71	186,18	1132,93	235,4	2	2	2	137,99
**IFN-γ**	2	2	17,28	2	2	45,27	2	2	2	2	2	2	4,28	40,09	124,34	30,56	2644,71	68,32	2	171,26	11,98	214,98
**TNF**	2	0,43	242,19	2	2	93,47	5,88	2	2	2	2	2	2	2	10,62	13,15	254,31	91,65	2	0	32,4	119,89
**IL-10**	2	2	73,3	2	2	11,36	2,85	2	36,08	2	0,44	2	510,92	820,8	14676,73	9431,91	17888,54	18087,62	256,07	1957,35	18907,83	18087,62
**IL-6**	19,52	118,92	3980,11	27,28	2	13162,3	7631,63	2	2	2	2	2	2	2	2	2	2	2	2	2	2	2
**IL-4**	2	2	2	2	2	2	2	2	2	2	2	2	2	2	0,28	2	2	1,93	2	2	2	2
**IL-2**	2	2	2	2	2	2	2	2	2	2	2	2	160,79	4,11	178,71	186,18	1132,93	235,4	2	2	2	137,99

Test Peptide 6 – Cytokines [pg/mL]

	**CT** (*n*=5)					**SC** (*n*=10)									
**IL-17a**	9,12	0,56	3,24	2	0,9	1	1	1	1	1	16,3	1	0,49	1	1
**IFN-γ**	2	2	9,57	53,46	121,32	5,43	15,44	39,46	12,54	48,84	864,67	7,8	27,96	11,46	1
**TNF**	2	2	0,01	68,32	4,89	59,52	50,8	27,51	31,97	115,95	63,64	6,7	8,61	21,76	1
**IL-10**	2	2	2	2	2	0,5	1	0,85	2,22	1	0,97	0,06	0,56	1,17	1
**IL-6**	2	2	88,15	28,36	156,22	682,28	184,41	414,28	493,99	457,31	1295,29	915,93	131,19	1937,71	937,12
**IL-4**	2	2	2	2	2	1	1	1	1	1	1	1	1	1	1
**IL-2**	2	2	2	19,96	14,36	4,76	16,73	9,39	5,25	7,02	8,73	3	15,56	3,73	1

Test Peptide 6 – Cytokines [pg/mL]

	**AD** (*n*=8)								**PT** (*n*=14)													
**IL-17a**	2	2	2	2	2	2	2	0,9	2	2	2	2	5,65	88,98	37,29	34,79	3958,92	97,39	0,16	75,92	17,56	78,3[illegible]
**IFN-γ**	2	2	12,09	2	2	212,02	2	2	2	2	2	2	2	0,37	0,17	2,76	484,98	88,7	2	2	50,33	59,9[illegible]
**TNF**	0,19	0,04	143,74	2	2	76,72	5,32	2	34,72	2	1,32	2	597,42	1123,46	3930,38	5875,04	18907,83	16558,63	336,41	1177,89	17305,17	17115[illegible]
**IL-10**	0	2	27,22	2	2	8,29	2,51	2	2	2	2	2	2	2	2	2	2	2	2	2	2	2
**IL-6**	37,14	108,99	2916,32	36,47	2	10722,58	6728,3	2	2	2	2	2	2	2	3,88	2	2	7,69	2	2	2	2
**IL-4**	2	2	2	2	2	2	2	2	2	2	2	2	5,65	88,98	37,29	34,79	3958,92	97,39	0,16	75,92	17,56	78,3[illegible]
**IL-2**	2	2	2	2	2	0,72	2	2	2	2	2	2	2	0,37	0,17	2,76	484,98	88,7	2	2	50,33	59,9[illegible]

Test Peptide 7 – Cytokines [pg/mL]

	**CT** (*n*=5)					**SC** (*n*=10)									
**IL-17a**	1,78	1,02	2	2	2	1	1	1	1	1	1	1	1	1	1
**IFN-γ**	2	2	107,88	190,8	480,61	33,13	17,01	141,93	12,27	78,04	552,45	9,27	9,02	18,33	1
**TNF**	2	2	0,97	6,11	30,93	179,79	50,22	86,32	239,9	154,54	85,4	8,9	6,33	8,61	0,4
**IL-10**	2	2	0,01	9,41	1,95	0,63	1	2,8	1,25	0,74	2,16	0,33	1	1,95	1
**IL-6**	2	2	243,43	42,7	1806,92	1563,4	312,13	2439,45	1760,69	958,82	5260,12	3007,92	207,4	5260,12	1846,92
**IL-4**	2	2	2	2	2	1	1	1	1	1	1	1	1	1	1
**IL-2**	2	2	2	18,66	42,69	15,79	20,86	16,26	8,08	5,64	1,9	2,07	4,29	5,25	1

Test Peptide 7 – Cytokines [pg/mL]

	**AD** (*n*=8)								**PT** (*n*=14)													
**IL-17a**	2	2	2	2	2	2	2	2	2	2	2	2	574,38	89,77	66,47	131,11	1202,75	186,18	29,18	136,82	56,39	27,19
**IFN-γ**	2	2	12,35	2	2	54,04	2	2	2	2	2	2	3,67	190,87	18,77	69,05	3032,26	70,53	4,89	95,41	12,35	10,77
**TNF**	2	2,93	326,46	2	2	101,47	5,99	2	2	2	2	2	0,28	4,3	0,33	21,16	101,04	119,89	2	2	55,28	52,14
**IL-10**	2	2	113,68	2	2	18,62	1,44	2	46,78	2	0,94	2	521,64	1942,64	2782,47	9532,93	18699,13	17115,24	538,08	1213,49	15502,31	16021,22
**IL-6**	25,7	205,65	4229,47	28,36	2	12201,31	5081,55	2	2	2	2	2	2	2	2	2	2	2	2	2	2	2
**IL-4**	2	2	2	2	2	2	2	2	2	2	2	2	1,07	2	0,49	2	2	0,79	2	2	2	2
**IL-2**	2	2	2	2	2	2	2	2	2	2	2	2	574,38	89,77	66,47	131,11	1202,75	186,18	29,18	136,82	56,39	27,19

Test Peptide 8 – Cytokines [pg/mL]

	**CT** (*n*=5)					**SC** (*n*=10)									
**IL-17a**	4,74	4,15	3,92	2	2	1	1	1	1,48	1	5,02	1	1	1	1
**IFN-γ**	2	2	20,5	206,94	2	7,21	10,28	19,89	1,64	9,4	14,1	1	1	1	1
**TNF**	2	2	0,8	82,54	8,17	2,17	5,41	12,1	5,09	3,73	11,73	1	1	0,35	1
**IL-10**	2	2	2	2	0,33	1	1	1,17	1,95	0,24	0,63	1	1	0,85	1
**IL-6**	2	117,77	428,71	25,56	1300,83	1508,94	379,59	564,25	781,13	610,01	905,53	1075,68	115,5	1389,58	697,79
**IL-4**	2	2	2	2	2	1	1	1	1	1	1	1	1	1	1
**IL-2**	2	2	2	8,02	2	0,46	2,71	6,17	2,61	0,28	9,39	1	1	1,42	1

Test Peptide 8 – Cytokines [pg/mL]

	**AD** (*n*=8)								**PT** (*n*=14)													
**IL-17a**	2	2	2	2	2	2	73,3	2	2	2	2	2	2	2	2	2	6,24	9,3	2	2	2	2
**IFN-γ**	2	2	1,96	2	2	10,17	2	2	2	2	2	2	184,58	115,67	3,34	58,19	1085,85	218,98	2	25,64	1,47	13,1
**TNF**	2	0,41	88,15	2	2	86,05	1,22	2	2	2	2	2	3,94	146,48	33,59	25,17	1696,17	87,85	2	53,35	2,68	4,89
**IL-10**	2	2	82,97	2	2	14,23	2	2	2	2	2	2	2	0,86	3,98	15,13	72,68	140,41	2	2	31,18	59,25
**IL-6**	14,31	133,61	2950,97	22,68	2	14517,28	4444,65	2	36,43	2	1,03	2	500,44	1964,96	8481,64	8849,04	16558,63	18289,06	9,07	1177,89	15502,31	14517,28
**IL-4**	2	2	2	2	2	2	2	2	2	2	2	2	2	2	2	2	2	2	2	2	2	2
**IL-2**	2	2	2	2	2	2	2	2	2	2	2	2	2	2	0,28	2	2	2	2	2	2	2

Test Peptide 9 – Cytokines [pg/mL]

	**CT** (*n*=5)					**SC** (*n*=10)									
**IL-17a**	1,78	1,11	3,03	2	2	1	1	1	0,69	1	1	1	3,41	0,1	1
**IFN-γ**	2	2	39,28	56,98	28,38	1	1	24,69	1	5,55	8,41	1	1	4,6	1
**TNF**	2	2	1,55	121,85	5,65	1	1	13,47	4,98	6,83	6,21	0,57	0,2	2,75	0,35
**IL-10**	2	2	2,45	2	2	1,47	1	0,6	3,31	2,38	1,05	0,12	1	1,75	1
**IL-6**	2	98,96	460,72	27,28	582,6	3538,76	292,51	1803,25	5125,29	5059,28	3450,9	2561,92	637,9	2792,48	1600,88
**IL-4**	2	2	2	2	2	1	1	1	1	1	1	1	1	1	1
**IL-2**	2	2	2	29,95	18,91	1	0,58	4,06	0,28	1	1	1	1	4,17	1

Test Peptide 9 – Cytokines [pg/mL]

	**AD** (*n*=8)								**PT** (*n*=14)													
**IL-17a**	2	2	2	2	2	2	118,02	2	2	2	2	2	2	0,65	2	2	16,58	0,25	2	2	2	2
**IFN-γ**	2	2	1,64	2	2	1,47	2	2	2	2	2	2	14,95	3,93	65,8	28,78	591,34	61,29	2	2	2	32,95
**TNF**	2	1,08	91,52	2	2	79,17	3,44	2	2	2	2	2	2,21	35,01	132,08	32,05	1840,71	55,12	1,03	89,69	5,76	107,88
**IL-10**	2	2	78,36	2	2	13,58	1,11	2	2	2	2	2	2	0,48	3,78	11,55	89,67	154,22	2	2	61,33	107,77
**IL-6**	19,71	159,26	2815	22,68	2	11193,23	4876,02	2	48,61	2	1,05	2	456	812,07	8044,83	7960,35	14359,68	18289,06	215,28	1313,82	15673,27	16377,4
**IL-4**	2	2	2	2	2	2	2	2	2	2	2	2	2	2	2	2	2	2	2	2	2	2
**IL-2**	2	2	2	2	2	2	2	2	2	2	2	2	2	2	6,01	2	2	0,22	2	2	2	2

Test Peptide 10 – Cytokines [pg/mL]

	**CT** (*n*=5)					**SC** (*n*=10)									
**IL-17a**	2	2	0,92	2	2	1	1	1	2,96	1,28	4,78	1	1	1	1
**IFN-γ**	2	4,3	37,5	29,59	866,43	13,24	14,54	26,83	24,89	78,7	32,09	3,47	7,68	8,04	1
**TNF**	2	0,37	3,21	344,16	46,22	64,34	15,83	30,8	51,97	127,26	89,13	12,48	3,06	10,5	0,48
**IL-10**	2	2	0,06	6,83	5,79	1,43	1	1,43	5,46	0,7	2,16	2,44	0,24	2,44	1
**IL-6**	2	126,04	383,08	522,26	3339,02	5469,62	396,54	1491,23	8479,15	2240	4225,75	6932,99	969,88	6932,99	2008,93
**IL-4**	2	2	2	2	2	1	1	1	1	1	1	0,1	0,1	1	1
**IL-2**	2	1,52	2	28,94	35,37	7,17	12,35	15,79	7,77	3,41	10,81	3,62	1,27	8,24	1

Test Peptide 10 – Cytokines [pg/mL]

	**AD** (*n*=8)								**PT** (*n*=14)													
**IL-17a**	2	2	2	2	2	2	2	5,41	2	2	2	2	2	2	2	2	0	4,2	2	2	2	2
**IFN-γ**	2	2	8,03	2	2	267,48	11,33	2	2	2	2	2	117,7	6,28	118,2	2	1132,93	667,2	313,49	2	14,32	76,99
**TNF**	2	5,51	183,77	2	2	225,83	11,28	2	2	2	2	2	3,94	28,22	114,67	4,09	3633,09	118,21	3,18	100,44	10,61	244,3
**IL-10**	2	2	57,75	2	2	17,83	0,92	2	2	2	2	2	2	2	7,39	2	398,18	125,08	2	2	63,47	125,08
**IL-6**	12,27	138,91	3657,45	41,19	2	13306	5577,74	2	64,2	2	0,66	2	428,71	803,43	9842,88	2566,74	16558,63	18289,06	582,6	1326,94	17691,79	17497,35
**IL-4**	2	2	2	2	2	2	2	2	2	2	2	2	2	2	2	2	2	2	2	2	2	2
**IL-2**	2	2	2	2	2	2	2	2	2	2	2	2	2	2	4,12	2	2	5,72	1,93	2	2	2

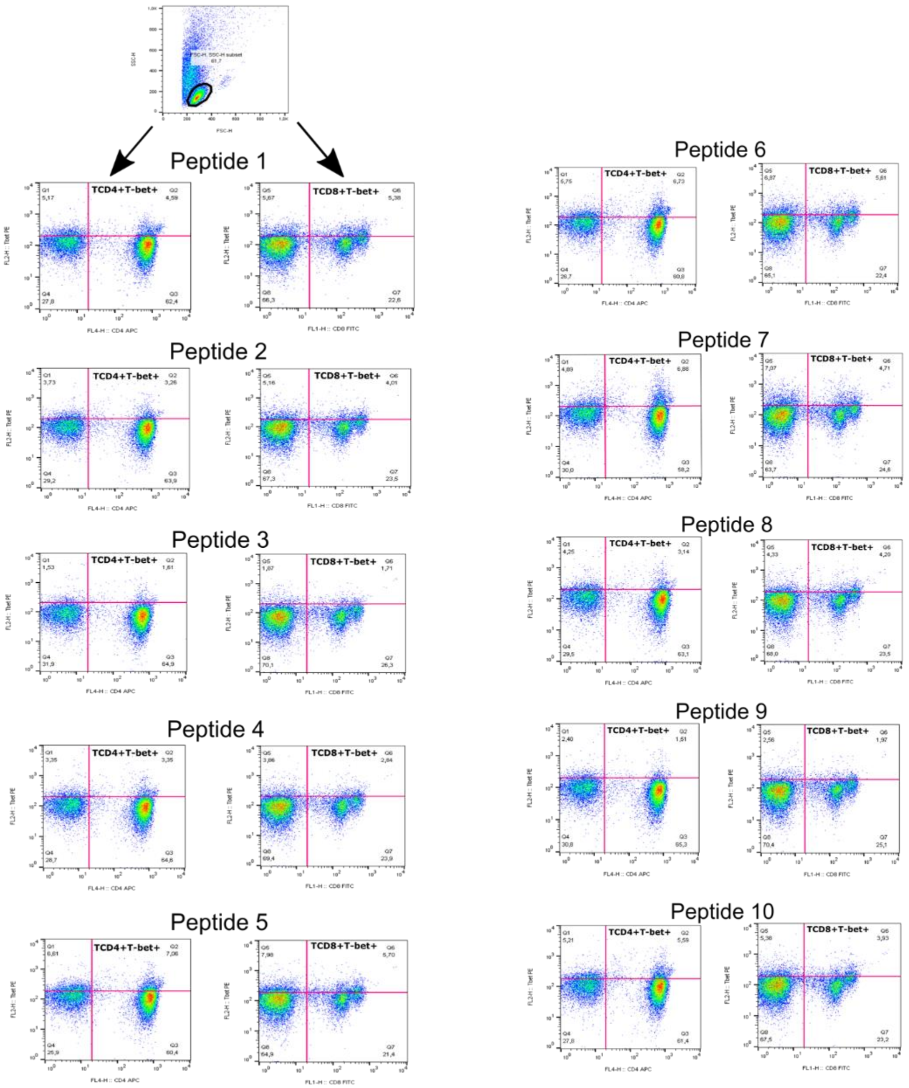

Suppl. Fig. S3: Intracellular flow cytometry staining for T-bet in human CD4$^+$ and CD8$^+$ T cells. Dot-plots are representative staining for intracellular T-bet in CD4$^+$ and CD8$^+$ T cells from one PT individual.
